# Choroidal microvasculature dropout is spatially associated with optic nerve head microvasculature loss in open-angle glaucoma

**DOI:** 10.1038/s41598-021-94755-8

**Published:** 2021-07-26

**Authors:** Min Kyung Song, Joong Won Shin, Jin Yeong Lee, Ji Wook Hong, Michael S. Kook

**Affiliations:** Department of Ophthalmology, University of Ulsan, College of Medicine, Asan Medical Center, 88, Olympic-Ro 43-Gil, Songpa-Gu, Seoul, 05505 Korea

**Keywords:** Biomarkers, Diseases

## Abstract

The presence of parapapillary choroidal microvasculature dropout (CMvD) may affect optic nerve head (ONH) perfusion in glaucoma patients, since parapapillary choroidal vessels provide vascular supply to the neighboring ONH. However, it remains to be determined whether the presence of parapapillary CMvD is associated with diminished perfusion in the nearby ONH. The present study investigated the spatial relationship between CMvD and ONH vessel density (ONH-VD) loss in open-angle glaucoma (OAG) eyes using optical coherence tomography angiography (OCT-A). This study included 48 OAG eyes with a single localized CMvD confined to the inferotemporal parapapillary sector and 48 OAG eyes without CMvD, matched for demographic and ocular characteristics. Global and regional ONH-VD values were compared between eyes with and without CMvD. The relationships between ONH-VD outcomes and clinical variables were assessed. ONH-VDs at the inferotemporal ONH sectors corresponding to the CMvD location were significantly lower in eyes with compared to those without CMvD. Multivariable linear regression analyses indicated that a lower inferotemporal ONH-VD was independently associated with CMvD presence and a greater CMvD angular extent (both *P* < 0.05). The localized presence of parapapillary CMvD in OAG eyes is significantly associated with ONH-VD loss in the neighboring ONH location, with a spatial correlation.

## Introduction

Ocular blood flow in the optic nerve head (ONH) and parapapillary retina is thought to have a pathogenic role in the development and progression of glaucomatous optic neuropathy^1–3^. The parapapillary choroid represents an anatomical region surrounding the prelaminar tissue of the ONH. The choroidal microvasculature within this parapapillary area is of great interest, since parapapillary choroidal vessels perfuse the ONH through the provision of centripetal vascular networks to the laminar cribrosa (LC), as well as to the prelaminar tissue within the ONH, after they receive a blood supply from the short posterior ciliary arteries (SPCAs)^4,5^. Hence, a choroidal vascular insufficiency may indicate a compromise in ONH perfusion, which may play an important role in the pathogenesis of open-angle glaucoma (OAG).

The recent introduction of optical coherence tomography angiography (OCT-A) has led to numerous subsequent studies on an entity known as choroidal microvasculature dropout (CMvD), which is defined as a focal complete loss of microvasculature in the choroidal layer within the β-zone parapapillary atrophy (β-PPA) area in OAG eyes^6–9^. According to these studies, CMvD has been predominantly detected at the inferior hemiretinae in glaucomatous eyes, while being associated with glaucomatous visual field (VF) damage confined to the superior hemifield at initial presentation^6–8^. Moreover, the presence of CMvD may represent parapapillary choroidal vascular impairment, as suggested by a recent study showing that an indocyanine green angiography (ICGA)-determined perfusion defect was compatible with an OCT-A determined microvasculature dropout^9^. Since ONH perfusion may be dependent on adjacent parapapillary choroidal circulation^4,5^, the presence of CMvD may be regarded as a clue for compromised perfusion in the ONH of OAG eyes. Despite these inferences however, little is known about the spatial relationship between the presence of CMvD and ONH microcirculation in glaucoma. We hypothesized that the detection of CMvD in OAG eyes may be associated with ONH vessel density (ONH-VD) loss with a spatial correlation. The aim of this study was to determine quantitatively whether a localized CMvD at the inferior retinae is topographically associated with a greater amount of ONH-VD loss in eyes with CMvD [CMvD(+)], when compared with eyes without CMvD [CMvD(−)], matched for glaucoma disease severity, including VF mean deviation (MD), average retinal nerve fiber layer thickness (RNFLT) and average circumpapillary vessel density (cpVD) using OCT-A.

## Results

Of the 150 eyes from the 150 OAG subjects that initially met the eligibility criteria for this study, 10 (6.6%) and 14 (9.3%) eyes were excluded owing to a poor OCT-A clarity and a focal weak signal that prevented a proper CMvD evaluation or ONH-VD quantification, respectively. Thirteen (8.7%) and 17 (11.3%) eyes were also excluded due to a poor horizontal B-scan image and pseudo-ONH-MvD, respectively. Hence, 96 eyes from 96 OAG patients met our final inclusion criteria, including 48 eyes with CMvD and 48 eyes without CMvD after 1:1 propensity score matching. Inter-examiner agreements regarding the determination of the presence of CMvD and ONH-MvD were excellent (k = 0.915; *P* < 0.001, k = 0.935; *P* < 0.001, respectively). The inter-examiner intraclass correlation coefficient (ICC) for measurement of the CMvD angular extent was 0.984 (95% CI 0.977–0.989; *P* < 0.001).

Comparisons of the demographic and clinical characteristics between the CMvD(+) and CMvD(−) groups are presented in Table 1. There were no significant between-group differences in age, sex, spherical equivalent (SE), AL, central corneal thickness (CCT), mean deviation (MD), baseline intraocular pressure (IOP), systolic blood pressure (SBP), diastolic blood pressure (DBP), mean ocular perfusion pressure (MOPP), average retinal nerve fiber layer thickness (RNFLT), superior RNFLT, average circumpapillary vessel density (cpVD), or superior cpVD. However, the CMvD(+) patients showed significantly lower inferior RNFLT (*P* = 0.016) and inferior cpVD (*P* = 0.006) compared to the CMvD(−) patients. Table 2 shows that the global and inferotemporal ONH VD were significantly lower in the CMvD(+) group than in the CMvD(−) group (*P* = 0.004 and *P* = 0.013, respectively). The presence of ONH-MvD was observed only in the CMvD(+) eyes and the difference in the proportions was statistically significant (*P* < 0.001). The CMvD(+) patients exhibited significantly lower global pCVD compared to the CMvD(−) patients (*P* < 0.001).

**Table 1:** Clinical demographics of study eyes with open-angle glaucoma with (+) or without (−) choroidal microvasculature dropout (CMvD).

	CMvD(−) (n = 48)	CMvD(+) (n = 48)	P value
Age	56.5 ± 11.2	55.6 ± 10.4	0.707
Sex (M:F)	30:18	21:27	0.066
SE	− 2.80 ± 3.2	− 2.40 ± 2.9	0.517
AXL	25.36 ± 1.2	25.36 ± 1.4	0.996
CCT	537.8 ± 39.8	543.2 ± 36.7	0.502
MD	− 6.35 ± 3.3	− 6.58 ± 3.6	0.745
Baseline IOP	15.0 ± 4.4	15.6 ± 3.9	0.467
SBP	124.8 ± 14.0	122.9 ± 12.1	0.546
DBP	76.3 ± 8.7	74.9 ± 9.0	0.501
MOPP	51.6 ± 6.0	50.6 ± 6.5	0.511
Average RNFLT	74.18 ± 9.4	70.71 ± 10.3	0.084
Superior RNFLT	97.93 ± 20.5	94.18 ± 21.2	0.384
Inferior RNFLT	74.93 ± 15.3	68.14 ± 11.4	0.016*
Average cpVD	43.39 ± 4.9	41.73 ± 5.2	0.114
Superior cpVD	45.58 ± 5.4	45.10 ± 6.5	0.703
Inferior cpVD	41.20 ± 5.7	38.10 ± 5.1	0.006*

Data are reported as a mean ± standard deviation.

*CMvD* choroidal microvasculature dropout, *M* male, *F* female, *AXL* axial length, *MD* mean deviation, *IOP* intraocular pressure, *SBP* systolic blood pressure, *DBP* diastolic blood pressure, *MOPP* mean ocular perfusion pressure, *RNFLT* retinal nerve fiber layer thickness, *cpVD* circumpapillary vessel density, *ONH VD* optic nerve head vessel density, *pCVD* parapapillary choroidal vessel density, *ONH-MvD* optic nerve head microvasculature dropout.

*P < 0.05 by unpaired t-test unless otherwise indicated.

**Chi-square test.

**Table 2:** Optic nerve head and parapapillary choroidal vessel density measurements in eyes with open-angle glaucoma with (+) or without (−) choroidal microvasculature dropout (CMvD).

	CMvD(−) (n = 48)	CMvD(+) (n = 48)	P value
Global ONH VD	61.92 ± 6.0	58.14 ± 6.6	0.004*
ONH VD superior	62.90 ± 8.0	61.61 ± 7.5	0.415
ONH VD inferior	62.29 ± 6.9	57.81 ± 10.0	0.013*
ONH-MvD (+/−)	0/48 (0%)	23/48 (47.9%)	< 0.001**
ONH-MvD:superior (+/−)	0/48 (0%)	1/48 (2.1%)	0.315**
ONH-MvD:inferior (+/−)	0/48 (0%)	22/48 (45.8%)	< 0.001**
Global pCVD	76.17 ± 5.3	70.15 ± 6.3	< 0.001*

Data are reported as a mean ± standard deviation.

*ONH* optic nerve head, *VD* vessel density, *ONH VD* optic nerve head vessel density, *pCVD* parapapillary choroidal vessel density, *ONH-MvD* optic nerve head microvasculature dropout.

*P < 0.05 by unpaired t-test unless otherwise indicated.

**Chi-square test.

Based on the univariable linear regression analyses, the global ONH-VD was found to be significantly associated with a female gender (β = 3.182, *P* = 0.014), SE (β = − 0.444, *P* = 0.043), inferior cpVD (β = 0.238, *P* = 0.046), and the presence of CMvD (β = − 3.784, *P* = 0.004). By multivariable analysis, the presence of CMvD (β = − 4.007, *P* = 0.005) remained significantly associated with global ONH-VD. By univariable linear regression analyses, a female gender (β = 3.527, *P* = 0.043), the presence of CMvD (β = − 4.483, *P* = 0.013) and CMvD angular extent (β = − 0.208, *P* = 0.049) were found to be significantly associated with inferotemporal ONH VD. In two separate multivariable analyses, which were conducted since there were no data for the CMvD angular extent in eyes without CMvD, the CMvD angular extent (β = − 0.232, *P* = 0.031) and the presence of CMvD (β = − 3.690, *P* = 0.041) showed a significant association with the inferotemporal ONH-VD.

In the univariable logistic regression analyses, inferior RNFLT (odds ratio [OR] 0.962; *P* = 0.020), inferior cpVD (OR 0.900, *P* = 0.008), parapapillary choroidal vessel density (pCVD, OR 0.842, *P* < 0.001), ONH VD (OR 0.907, *P* = 0.007), and inferior ONH VD (OR 0.939, *P* = 0.017) showed a significance association with the presence of CMvD, with *P* values less than 0.05. In two separate sets of multivariable analyses to avoid multicollinearity between global and sectoral ONH-VD values, pCVD (OR 0.831, *P* < 0.001; model 1), ONH-VD (OR 0.898, *P* = 0.006; model 1), and inferior ONH-VD (OR 0.938, *P* = 0.023; model 2) had significant associations with the presence of CMvD.

**Figure 1:**
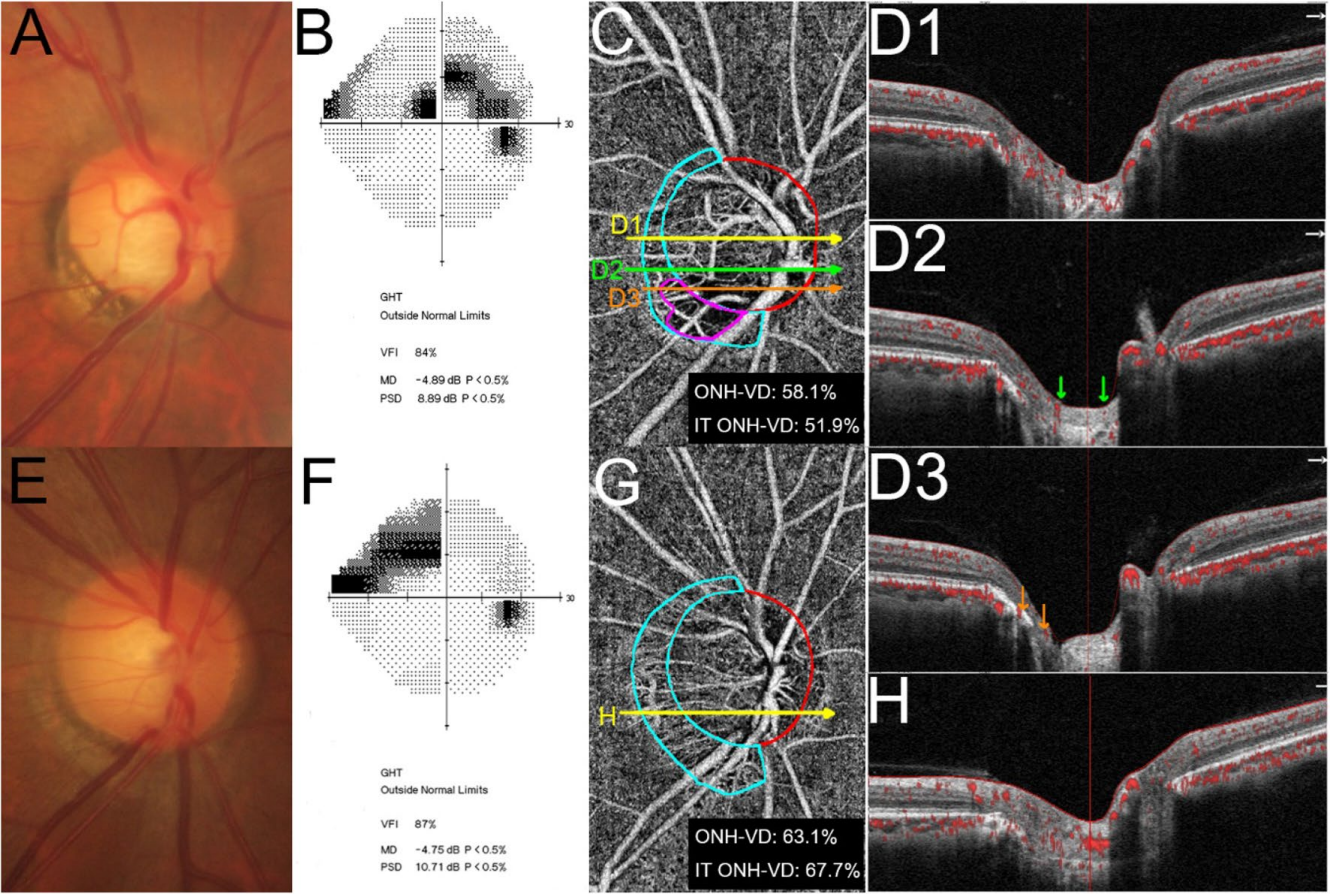
Representative eyes with and without choroidal microvasculature dropout (CMvD) having superior hemifield visual field (VF) loss. (**A**–**D**) A 53-year-old male with open-angle glaucoma (OAG) and CMvD in his right eye. (**E**–**H**) A 51-year-old male with OAG without CMvD in his right eye. Both eyes were similar in terms of age, gender, and VF severity. The CMvD(+) eye showed inferotemporal (IT) neural rim thinning (**A**) and superior paracentral VF loss with mean deviation (MD) of − 4.89 dB (**B**). In optical coherence tomography angiography (OCT-A) en-face image of the optic nerve head (ONH), loss of IT ONH vessel density (ONH-VD) adjacent to the CMvD location was observed with IT ONH-VD measured to be 51.9% (**C**). A horizontal B-scan revealed an intact microvasculature signal in the prelaminar tissue and anterior portion of lamina cribrosa (LC) where there was no ONH-VD loss (**D1**). In contrast, another horizontal B-scan showed loss of microvasculature signal in the prelaminar tissue and anterior portion of LC in the area between the 2 green arrows where there was ONH-VD loss (**D2**). Another horizontal B-scan showed that the loss of a microvasculature signal was also observed in the choroidal layer between the 2 orange arrows where there was a CMvD next to the presence of ONH-VD loss (**D3**). The red ellipse and sky blue lines indicate the ONH and β-zone parapapillary atrophy margin. The CMvD area is enclosed by the purple colored border. The CMvD(−) eye also showed IT neural rim thinning (**E**) and superior peripheral VF loss with MD of − 4.75 dB (**F**). In OCT-A en-face image of the ONH, an intact ONH-VD was observed in the IT ONH location with IT ONH-VD measured to be 67.7% (**G**) and intact microvasculature signal in the prelaminar tissue and anterior portion of the LC (**H**). All OCT-A images were created by Avanti system version 2018.1.0.37.

Figure 1 shows representative OAG eyes with and without CMvD.

## Discussion

In this current study we used OCT-A to evaluate the spatial relationship between the presence of CMvD at an inferotemporal location and ONH-VD loss in OAG eyes. We found that global and inferotemporal ONH-VDs were significantly lower in eyes with CMvD than in eyes without CMvD, despite all demographic characteristics, including the age profile, AL, and severity of glaucomatous damage, being matched in both patient groups using propensity score matching method. Moreover, the inferotemporal ONH-VD consistently showed a significant association with the presence of CMvD at the inferior hemiretinal location and CMvD angular extent in our current series of OAG eyes. These findings were further confirmed by logistic regression analyses, which indicated that inferotemporal ONH-VD loss was an independent predictor of the presence of CMvD based on the results of multivariable analyses. Our current findings thus suggest that CMvD could be an important clinical sign of regional microvascular insufficiency in the ONH, with a spatial correlation in OAG eyes.

It is well established that CMvDs most often occur at the inferotemporal parapapillary region (i.e., at the 7 o’clock position) of the choroid within the β-PPA^8,10,11^. Although it is unclear why CMvD is frequently found at the inferotemporal parapapillary region, this location is consistent with the macular vulnerability zone in the retina, which corresponds to the zone that is particularly susceptible to glaucomatous damage^12^. In line with this possibility, previous studies have shown that a localized CMvD at the inferior hemiretinae in OAG eyes is associated with glaucomatous RNFL damage in the same hemiretinae, and VF damage in the superior hemifield of eyes^8,13^. These earlier findings suggested that the presence of CMvD may play an important role in the pathogenesis of glaucoma, the mechanism of which remains to be further explored. With this in mind, OAG eyes with a single localized CMvD at the inferior hemiretinae were enrolled in our current study with the aim to investigate the topographic relationship between the presence of CMvD and regional ONH microvasculature loss.

Our present study revealed that the RNFLT and cpVD are significantly lower in the CMvD-affected hemiretinae (i.e. inferior hemiretinae) of CMvD(+) eyes than in those of CMvD(−) eyes, notwithstanding a similar average RNFLT and VF severity in both groups (*P* < 0.05, Table 1). These findings are consistent with those of Jo et al., which demonstrated that OAG eyes with a localized CMvD exhibit more severe RNFLT and cpVD loss in the same location compared to those without CMvD despite having similar glaucoma severity^13^. Furthermore, the global pCVD in the β-PPA was significantly lower in CMvD(+) eyes than CMvD(-) eyes in the current series of OAG eyes (Table 2). These results are also supported by our recent report, showing that OAG eyes with a localized CMvD exhibit more generalized pCVD attenuation in the β-PPA area compared to those without CMvD, leading to a greater reduction in the pCVD, despite having similar glaucoma severity^14^.

SPCAs may provide vascular networks to the prelaminar tissue and LC of the ONH^4,5^. However, the role of parapapillary choroidal circulation in ONH perfusion has been controversial. Some reports have indicated that it is a major source of the blood supply to the prelaminar region or LC of the ONH^4,5^, whereas another study suggested it plays a minimal role only in this regard^15^. Recently, Lee et al. investigated perfusion within and around the ONH using ICGA, and found that the parapapillary choroidal arteriole was universally filled before the adjacent ONH filling, thereby postulating that the parapapillary choroid may be an important source of perfusion in the anterior ONH^16^. Accordingly, the presence of CMvD, which represents perfusion impairment in the parapapillary choroidal layer, may lead to perfusion reduction in the neighboring area of the ONH. Nonetheless, this possibility has not been fully elucidated to date.

Our present study confirms that CMvD(+) eyes show a greater reduction of inferotemporal ONH-VD, with a spatial correlation, than CMvD(−) counterparts, despite having similar demographic and ocular characteristics (Table 2). Moreover, ONH-MvD was observed only in the CMvD(+) group in our present analyses. This result was consistent with the findings of a previous report, which demonstrated a 10-fold higher risk of ONH-MvD in eyes with CMvD^17^. OAG eyes with CMvD have previously displayed a poorer disease prognosis, including a more rapid rate of both RNFL thinning and central VF progression, suggesting that CMvD may have prognostic value^18,19^. However, one limitation of CMvD detection is that its presence cannot be evaluated in eyes without β-PPA. Since CMvD and ONH-MvD are strongly correlated in terms of their coexistence as noted in our study, the presence of ONH-MvD may be a marker for glaucoma or its progression in eyes without β-PPA. Of interest in this regard, a previous study evaluating ONH whole image VD using OCT-A has reported that a low baseline ONH-VD is significantly associated with a faster rate of RNFL progression^20^. Prospective longitudinal studies are needed in the future to elucidate the temporal relationship between ONH-VD loss or the presence of ONH-MvD and subsequent glaucoma progression.

Of all subjects initially included in this study, 11.3% of the eyes had pseudo-ONH-MvD. This percentage was lower than that of the study by Akagi et al. (30.1%)^17^. The discrepancy between the two studies could result from the different study populations (e.g., Korean vs. Caucasian) and selection biases. In particular, many subjects of this study had myopic refractive error with an average AXL of 25.36 mm, which results in a higher ratio of the large optic disc^21,22^. It is therefore possible that the eyes in our study had better light penetration, so that the anterior LC border was better visualized. Moreover, glaucomatous myopic eyes have thinner neuroretinal rim^23^ which may minimize shadowing effect of neuroretinal rim on anterior LC border.

Our current multivariable analyses revealed that global ONH-VD, excluding the VD of the large projecting vessels from optic disc, is independently associated with the presence of CMvD. Moreover, the regional inferotemporal ONH-VD value was significantly associated with the presence and angular extent of CMvD. These findings are not surprising considering that the parapapillary choroidal microvasculature may be supplied by the PCAs^4,5^, which also provide the blood supply to the ONH including the prelaminar tissue and LC. Thus, CMvD or other signs of compromise in the parapapillary choroidal perfusion may also indicate a vascular insufficiency at nearby ONH structures.

Logistic regression analyses in our present study indicated that lower global and inferotemporal ONH-VD values were found to be significant predictors of the presence of CMvD. Because the parapapillary choroidal vascular networks are extensively interconnected with other distant vessels in the ONH^15,24^, the presence of a localized CMvD may be associated not only with regional ONH vascular loss, but also indicate more widespread vascular insufficiency to the ONH in OAG eyes. This may explain our current findings. In addition, our present study shows that global pCVD is a significant independent variable associated with the presence of CMvD in eyes with OAG. This finding is consistent with the results of our recent study, which demonstrated that a localized CMvD is associated with a generalized reduction in the pCVD throughout the ß-PPA zone, as measured by OCT-A^14^.

There were several limitations of our present study of note. First, both patient groups were matched retrospectively rather than selected prospectively through population-based screening, which could have introduced selection bias. However, we attempted to overcome this limitation by performing propensity score matching according to glaucoma severity, including VF MD, average RNFLT, and average cpVD, in the comparison of the ONH-VD and ONH-MvD of CMvD(+) eyes with those of CMvD(−) eyes. Another limitation of our study was that we did not evaluate the presence of focal LC defect, which is one of most important factors related to both CMvD and ONH-MvD^17^. Future studies are needed using swept-source OCT with a high resolution suitable for evaluating focal LC defects, in which two groups can be matched in terms of the presence of focal LC defect. Another source of selection bias is that our patients were recruited from a single tertiary university-based practice. Hence, the characteristics of our patient cohort may differ from those in the general population. Second, subjectivity might have affected the detection of CMvD/ONH-MvD, the manual marking of the region of interest (ROI) for ONH-VD and pCVD measurement, and measurements of CMvD angular extent because of the inherent limitations associated with current OCT-A imaging. We attempted however to minimize any measurement errors related to subjectivity by having two independent examiners determine the presence of CMvD/ONH-MvD, with discrepancies adjudicated by a third examiner, an approach that resulted in excellent inter-examiner agreement (k = 0.915 and 0.935; *P* < 0.001, respectively). The inter-examiner ICC for measurements of the CMvD angular extent was 0.984 (95% CI 0.977–0.989; *P* < 0.001). Third, microvasculature evaluations within ONH in the current study were performed using whole-signal mode OCT-A images, which are subject to various artifacts. In doing so, only images of a well-visualized ONH were included in our analysis to remove or minimize the potential effects of such artifacts (e.g., large retinal vessel projection or neural rim shadowing). For the identification of ONH-MvD, only eyes with a well-visualized anterior LC in the horizontal B-scans were included to confirm the complete loss of a microvasculature throughout the anterior LC^17^. Hence, eyes with relatively large discs, shallow cupping, or a thin neural rim could have been preferentially selected for better anterior LC visualization in our present series. Fourth, the ONH-VD was assessed quantitatively only in the temporal side of the ONH. This is because deeper structures of the ONH in the nasal side are not easily visualized using OCT-A due to large retinal vessels and a thick neural rim, especially in myopic eyes with an optic disc tilt^25,26^ Fifth, we evaluated the pCVD and CMvD using the ImageJ software (version 1.52; Wayne Rasband, National Institutes of Health, Bethesda, MD, USA) since there is currently no commercially available software to automatically assess VD in the deep layer of the ONH and parapapillary region. Sixth, there was no healthy control group to determine whether CMvD or ONH-VD occurs in this group and compare pCVD and ONH-VD values between the control and OAG groups in the present study. However, to this date, CMvD has not been reported to occur in the healthy control eyes in the literature^8,13^. Finally, because of the cross-sectional design of our present study, the longitudinal relationship between the detection of CMvD and the emergence of ONH-MvD or a reduced ONH-VD in the neighboring ONH location could not be explored in our analysis.

In conclusion, the presence of a CMvD at an inferotemporal location is spatially associated with ONH-VD reduction, as quantitatively measured by OCT-A, in eyes with OAG. The presence of a localized CMvD may predict a diminished perfusion in the neighboring location of the ONH. Because CMvD is closely associated with ONH hypoperfusion, with a spatial correlation, determination of this association may enable risk profiling for CMvD in glaucoma management, as ONH hypoperfusion may contribute to greater disease severity at presentation and a poorer prognosis during follow-up.

## Methods

### Subjects

The present study was a retrospective study of consecutive OAG patients who visited the glaucoma service of Asan Medical Center, Seoul, Korea, from November 2016 to December 2019. The Institutional Review Board (IRB) of Asan Medical Center approved the study protocols, and all procedures followed the principles of Declaration of Helsinki. The requirement for informed consent from the patient subjects was waived by the IRB of Asan Medical Center due to the retrospective nature of the analyses.

Initially, all patients underwent a comprehensive ophthalmic examination, including slit-lamp biomicroscopy, Goldmann applanation tonometry, and gonioscopy. AL was measured with an IOL master (Carl Zeiss Meditec, Dublin, CA) and the CCT was determined by ultrasound pachymetry (DGH-550; DGH Technology, Inc., Exton, PA). Patients also underwent dilated color fundus photography (Canon, Tokyo, Japan), ONH stereoscopic photography, red-free RNFL photography (Canon), Humphrey field analyzer Swedish Interactive Threshold Algorithm (SITA) 24-2 VF testing (Carl Zeiss Meditec), Cirrus spectral-domain optical coherence tomography (SD-OCT; Carl Zeiss Meditec; version 6.0) and OCT-A (AngioVue; Optovue, Inc., Fremont, CA). SBP and DBP measurements were taken during the initial work-up in the outpatient clinic. The MOPP was estimated as the difference between 2/3 of the mean arterial pressure (MAP) and IOP.

For inclusion in the current study, all patients were required to meet the following criteria: aged > 18 years; a glaucomatous-appearing ONH regardless of the IOP level (i.e., focal or generalized narrowing or disappearance of the neuroretinal rim, an optic disc hemorrhage [ODH], vertical cup-to-disc asymmetry > 0.2 not explained by optic disc size, or an RNFL defect); a visible β-PPA on fundus photography; open anterior chamber angles on gonioscopy; and glaucomatous VF defects confined to the superior hemifield, corresponding to ONH appearance^14^. Eyes with glaucomatous VF defects confined to the superior hemifield were defined as those with (1) three or more adjacent points with *P* < 0.05 on a pattern deviation (PD) probability map, or with two or more test points with *P* < 0.02 or smaller on a PD probability map in the superior hemifield; (2) no clusters of three points with *P* < 0.05 or two points with *P* < 0.02 on both the total deviation and PD probability maps in the inferior hemifield; and (3) a glaucoma hemifield test (GHT) result outside normal limits^27^. Glaucomatous VF was confirmed on two consecutive reliable SITA 24–2 VF tests. The second perimetric result was used to minimize the learning effects.

OAG subjects that met our inclusion criteria were assigned into two groups (case vs. control) according to the presence of CMvD. Eyes with a localized CMvD confined to the inferotemporal sector of the parapapillary choroid within the β-PPA were consecutively selected. OAG eyes without CMvD were selected from the same database after 1:1 matching to CMvD(+) eyes with regards to clinical and ocular demographics to minimize the confounding effects of these variables on the ONH-VD evaluation in each group.

Exclusion criteria for our study subjects included cataracts of more than C2, N2, or P2 based on the Lens Opacities Classification System III^28^; severe myopic ONH or fundus changes (i.e. posterior staphyloma) that may impair adequate ONH/macular/VF evaluation for glaucoma; a history of any intraocular surgery or laser procedure; or a history of other ophthalmic or neurologic diseases that could affect VF test or ONH/retinal structural integrity, including age-related macular degeneration, diabetic retinopathy, and retinal vascular occlusive diseases. Eyes with unreliable VF results (fixation loss > 20%, false-positive error > 15%, and false-negative error > 15%) were also excluded. One eye was randomly selected if both eyes were eligible for inclusion.

### Superficial retinal vessel density measurement

All subjects underwent OCT-A, which uses a split-spectrum amplitude-decorrelation angiography algorithm to assess the dynamic motion of red blood cells and presents a three-dimensional angiogram of perfused retinal vasculature^29^. The AngioVue OCT-A generates en-face images and vascular data at various user-defined retinal layers via an automated layer segmentation algorithm around the ONH^29^. In the current study, all OCT-A images were analyzed using Avanti system version 2018.1.0.37.

The VD within the RNFL, which extends from the inner limiting membrane (ILM) to the posterior margin of the RNFL, on a 4.5 × 4.5 mm volumetric scan centered on the ONH was used to measure the cpVD as previously reported^29,30^. The average cpVD was automatically calculated in the RNFL using AngioVue OCT-A within a region defined as a 1000-µm-wide elliptical annulus extending from the optic disc boundary. The circumpapillary large vessels were automatically removed using built-in OCT-A software. Two 180° regional measurements of the cpVD (superior, inferior), automatically provided by the device, represented the corresponding hemiretinal values for this parameter.

### ONH and parapapillary choroidal vessel density measurement

**Figure 2:**
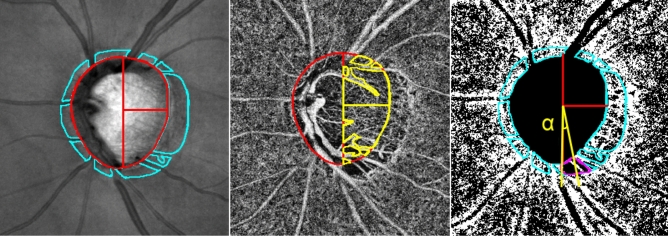
Measurements of optic nerve head vessel density (ONH-VD) and parapapillary choroidal vessel density (pCVD) and angular extent of choroidal microvasculature dropout (CMvD) in open-angle glaucoma (OAG) eye with CMvD using optical coherence tomography angiography (OCT-A). (Left) The red ellipse indicates the optic nerve head (ONH) margin, while the red lines denote the boundaries of ONH-VD measurement areas within the ONH. The sky blue outline indicates the β-parapapillary atrophy (β-PPA) margins, excluding large retinal vessels, identified on a scanning laser ophthalmoscopy (SLO) image, which are applied to same position within the β-PPA on the OCT-A choroidal layer en-face image. (Middle) The region of interest (ROI) within the ONH, excluding large retinal vessels, were manually demarcated (yellow outline). These area of interest were used to measure the global ONH-VD using ImageJ software. Superotemporal and inferotemporal ONH area divided by horizontal line were used for superior and inferior ONH-VD measurement, respectively. (Right) The selected area of interest, excluding large retinal vessels, within the β-PPA was converted to ROI, then applied to the 8- bit binary slab of OCT-A choroidal layer en-face image. The CMvD area is enclosed by the purple-colored border. The two points at which the circumferential border of the CMvD met the ONH margin were identified as the circumferential margins. The two yellow lines connect the ONH center to the circumferential margins of the CMvD. Angle α is the angular extent (degrees) of the CMvD. All OCT-A images were created by Avanti system version 2018.1.0.37.

ONH-VDs were evaluated on the ONH en-face image using the OCT-A whole-signal mode image, which is constructed from all OCT-A signals below the ILM. This method of evaluating ONH-VD has been previously validated. This method of evaluating choroidal and ONH vessel density using each image has been previously validated^17^. Briefly, the ONH boundary was defined manually as the inner margin of the peripapillary scleral ring identified on scanning laser ophthalmoscopy (SLO) images. This boundary was applied to the same position of ONH on the ONH en-face images using ImageJ software (Fig. 2, left, red ellipse)^17,31^. The center of the ONH was determined using ImageJ software. The temporal side of the vertical line passing through the ONH center was used for the analysis since the visualization of deep structures of ONH using OCT-A is limited at the nasal side of the optic disc due to large vessels and thick neural rim (Fig. 2, left, red line)^17,25,26^. For measurement of ONH-VD, ROI was marked manually excluding large projecting vessels within temporal side of ONH (Fig. 2, middle, yellow line).

pCVDs were evaluated on the 4.5 × 4.5 mm ONH choroidal layer en-face projection image produced by layer segmentation of signals from the retinal pigment epithelium to the inner border of sclera^6,11,14^. For the measurements of pCVD within the β-PPA, the β-PPA was marked as a ROI whilst excluding large projecting vessels wider than 3 pixels (approximately ≥ 33 μm) from the overlying retinal layer within the β-PPA on SLO images (Fig. 1, left, sky line)^14,32,33^. This ROI was applied to the same position within the β-PPA on the choroidal en-face image using ImageJ software.

An 8-bit binary slab was created based on the mean threshold algorithm of ImageJ software using ONH and choroidal layer en-face images^14^. This algorithm automatically measured the threshold as the average of the local grayscale distribution. The selected ROI for the ONH-VD and pCVD measurement was applied to the 8-bit binary slab of the en-face image (Fig. 2, middle, right). After assigning white pixels to vessels and black pixels to the background, VD was automatically calculated by ImageJ software as a percentage of vessel pixels within the ONH and β-PPA area relative to the total number of pixels within the ROI^14^.

### Choroidal and ONH microvasculature dropout assessment

In the present study, the CMvD was defined as a complete loss of the choriocapillaries and microvasculature without any visible microvasculature network within the β-PPA on OCT-A choroidal en-face imagery, and identified when the minimum angular width was greater than 200 µm or than the width of the central retinal vein^6,34^. The area of the CMvD was measured via ImageJ software after manually defining the CMvD margin (Fig. 2, right, purple line). The two points at which the circumferential borders of the CMvD met the optic disc margin were defined as the angular circumferential margins. The angular extent of CMvD was measured by drawing two lines connecting the center of optic disc to the circumferential margins of the CMvD on an OCT-A image using ImageJ software (Fig. 2, right, yellow line). This method of measuring the angular extent of the CMvD has been previously reported in various studies^13,35^.

The ONH microvasculature dropout (ONH-MvD) was defined as a complete loss of OCT-A signal within the ONH, which is 200 µm or more in width and 100 µm or more in length^17^. The ONH-MvD was also evaluated on the temporal side of the optic disc on the ONH en-face projection image. For the identification of an ONH-MvD, the visualization of the anterior LC was confirmed in all horizontal B-scan images at the area of complete loss of OCT-A signals within the ONH in order to verify the complete microvasculature loss throughout the deep layer of the ONH^17^. If the anterior LC portion was not clearly visualized at the area of complete loss of OCT-A signals in the horizontal B-scan images, the eyes were considered to have pseudo-ONH-MvD^17^, and were excluded from analysis.

All choroidal layer and ONH en-face projection images were evaluated independently by two co-authors of this study who are glaucoma specialists (M.K.S. and J.W.S.), who were blind to the clinical data for the patients. The presence of CMvD and ONH-MvD was also independently assessed by M.K.S. and J.W.S, with any discrepancies resolved by a third specialist (M.S.K.). The circumferential margin of the CMvD was again assessed independently M.K.S. and J.W.S, in which the angular extent determined by these two specialists was averaged and used in the final analysis to minimize inter-examiner variability. Eyes with poor quality OCT-A images were excluded, including those with a signal strength index (SSI) below 50, with a focal weak signal due to floater or posterior vitreous detachment, with a motion artifact visualized as an irregular vessel pattern or disc boundary on en-face images, or those with RNFL segmentation errors.

### Statistical analyses

In order to minimize the confounding effects on the ONH-VD and ONH-MvD assessment due to selection bias related to different study populations of the two groups, propensity score analysis match method was performed. This method can perform a quasi-randomized comparison from a retrospective study^36^. In this method, multiple logistic regression analysis was performed to determine the propensity score using the following covariates (independent variables: VF MD, average RNFLT, and average cpVD) in this study^37^. A 1:1 matching defines that 1 control unit with similar propensity score in the control group [CMvD(−)] was matched to a single unit in the case group [CMvD(+)].

Inter-examiner agreements regarding the presence of CMvD and ONH-MvD and the CMvD angular extent were assessed using Kappa statistics and ICCs. A normal distribution was confirmed using the Kolmogorov–Smirnov test. For comparisons between groups, the independent t test or the Mann–Whitney U test was performed for continuous variables, based on the normality test. Categorical variables were compared with the chi-square test. Clinical factors associated with global and inferior ONH-VD were identified by univariable linear regression analysis. Variables with a *P* value less than 0.1 in the univariable analyses were included as independent variables in the multivariable model using a backward elimination approach. Univariable and multivariable logistic regression analyses were performed to determine the clinical factors associated with the presence of CMvD. Univariable analyses included systemic and ocular demographic and clinical factors. Independent variables with a *P* value < 0.1 in the univariable model were entered to the multivariable model. All statistical analyses were conducted using Statistical Package for Social Science version 22.0 (SPSS, IBM Corp., Amonk, NY), with two-tailed P-values < 0.05 considered statistically significant.
